# Cryo-EM structure of anchorless RML prion reveals variations in shared motifs between distinct strains

**DOI:** 10.1038/s41467-022-30458-6

**Published:** 2022-07-13

**Authors:** Forrest Hoyt, Heidi G. Standke, Efrosini Artikis, Cindi L. Schwartz, Bryan Hansen, Kunpeng Li, Andrew G. Hughson, Matteo Manca, Olivia R. Thomas, Gregory J. Raymond, Brent Race, Gerald S. Baron, Byron Caughey, Allison Kraus

**Affiliations:** 1 Research Technologies Branch, Rocky Mountain Laboratories, National Institute of Allergy and Infectious Diseases, National Institutes of Health, Hamilton, MT 59840 USA; 2 Department of Pathology, Case Western Reserve University School of Medicine, Cleveland, OH USA; 3 Laboratory of Persistent Viral Diseases, Rocky Mountain Laboratories, National Institute of Allergy and Infectious Diseases, National Institutes of Health, Hamilton, MT 59840 USA; 4 Cleveland Center for Membrane and Structural Biology, Case Western Reserve University, Cleveland, OH USA

**Keywords:** Cryoelectron microscopy, Pathogens, Infectious diseases, Encephalopathy

## Abstract

Little is known about the structural basis of prion strains. Here we provide a high (3.0 Å) resolution cryo-electron microscopy-based structure of infectious brain-derived fibrils of the mouse anchorless RML scrapie strain which, like the recently determined hamster 263K strain, has a parallel in-register β-sheet-based core. Several structural motifs are shared between these ex vivo prion strains, including an amino-proximal steric zipper and three β-arches. However, detailed comparisons reveal variations in these shared structural topologies and other features. Unlike 263K and wildtype RML prions, the anchorless RML prions lack glycophosphatidylinositol anchors and are severely deficient in N-linked glycans. Nonetheless, the similarity of our anchorless RML structure to one reported for wildtype RML prion fibrils in an accompanying paper indicates that these post-translational modifications do not substantially alter the amyloid core conformation. This work demonstrates both common and divergent structural features of prion strains at the near-atomic level.

## Abstract (web summary)

The authors report the near-atomic structure of the ex vivo aRML prion fibril. The comparison between this structure and the previously solved 263K prion fibril shows that both common and divergent features characterize these prion strains and their respective conformational replication templates.

## Introduction

Prion strains propagate as distinct infectious scrapie PrP (PrP^Sc^) conformers that can take the form of amyloid fibrils. However, the detailed structures of these conformers and how they differ between strains have been poorly understood. We recently solved a near atomic cryo-electron microscopy (cryo-EM)-based structure of a highly infectious brain-derived prion strain, i.e., 263K hamster-adapted scrapie^1^. The core of this fibril has PrP monomers stacked in a parallel in-register intermolecular β-sheet (PIRIBS)-based architecture. This natural prion core is more than twice as large as those of synthetic recombinant PrP fibrils that are likely to be noninfectious or at least orders of magnitude less infectious^2–4^. Lower resolution data (~10 Å) for another brain-derived prion fibril, i.e., the mouse anchorless RML (aRML) strain, indicated a large core with a distinct fibrillar morphology, but did not allow localization of the polypeptide backbone^1^. The aRML strain provides a striking contrast to 263K. Whereas 263K PrP^Sc^ contains glycophosphatidylinositol (GPI) anchors and is heavily glycosylated, aRML PrP^Sc^ lacks GPI anchors and, consequently, is poorly glycosylated^5,6^. Accumulation of aRML PrP^Sc^ in the brain is predominantly in extracellular amyloid plaques^6,7^ whereas 263K is found mostly in diffuse deposits that are closely associated with cellular membranes. Although 263K fibrils have a PIRIBS architecture^1^, aRML fibrils have been inferred by others to have a fundamentally different 4-rung β-solenoid-based architecture^8,9^. Here we report that with methodological improvements we have now obtained cryo-EM data for aRML fibrils with sufficient resolution (3.0 Å) to determine a detailed structure of the amyloid core.

## Results

Silver-stained PAGE gels and Western blots of the specific aRML preparation that we used for cryo-EM have been reported previously^10^ and indicate high purity with respect to PrP content. Previous mass spectrometry analyses of such aRML fibril preparations detected PrP polypeptides inclusive of residues 81–231 within the proteinase K-resistant core^10^. Intracerebral inoculation of serial dilutions of the purified preparation ranging from 100 ng protein down to 10 pg into susceptible transgenic mice (tga20)^11^ (*n* = 4 per dilution) caused terminal prion disease requiring euthanasia in all recipients. The incubations of further dilutions are ongoing, but inoculations of 100 fg have so far required euthanasia of 3 mice (*n* = 6 at this dilution), indicating a specific infectivity of ≥~10^10^ LD_50_/mg. This level is higher than the ~10^9^ LD_50_/mg reported for purified wildtype RML prions using a cell rather than animal-based bioassay^12^.

Cryo-electron tomography was performed to determine handedness of the fibril twist. Analyses of 12 tomograms indicated that all aRML fibrils sufficiently isolated for tomographic analysis (*n* = 64) were left-handed (Supplemental Fig. 1). Accordingly, a left-handed twist was used in the model building described below. As was the case for 263K prion fibril preparations^1^, we also observed globules along the sides of some of the fibrils, as is the case for the example shown in Supplemental Fig. 1. However, ~70% of the fibrils lacked visible globules. The nature of the globules is unknown, but their presence on some of the aRML fibrils indicates that they are not dependent upon the presence of GPI anchors.

**Fig. 1:**
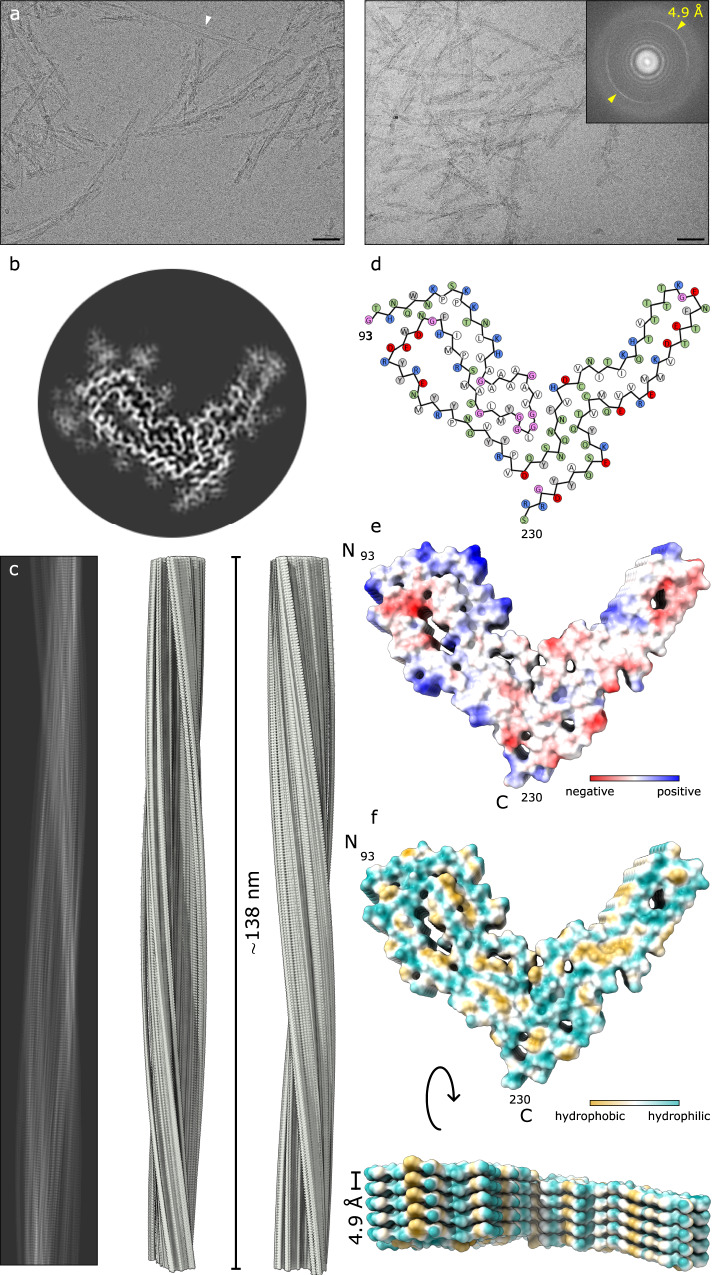
Cryo-EM-based structure of aRML fibrils. **a** 2D cryo-EM images of aRML fibrils. Bar = 50 nm. Inset depicts associated Fast Fourier transform showing signals from regular 4.9 Å spacings (yellow arrows). The white arrowhead indicates a rare example of aRML fibrils that appear to be paired. Micrographs shown are representative of 2272 movies collected for subsequent image processing and single particle analysis (see the “Methods” section). **b** Cross-sectional view of a density map projection. **c** Lateral view of the fibril density map with cross-over distance as indicated. **d** Core sequence showing relative orientations of side chains. Green, polar; blue, basic; red, acidic; white, aliphatic; gray, aromatic; pink—glycine. **e** Coulombic charge representation. **f** MLP hydrophobicity surface plot demonstrating interspersed hydrophobic interactions.

We obtained details of the structure of the aRML prion fibrils using single particle acquisition and helical reconstruction^13^ with parameters given in the “Methods” section and Supplemental Table. Most of the fibrils appeared to be composed of a single protofilament, but some seemed to be paired laterally (e.g., see Fig. 1a, arrowhead). Fast Fourier transforms of particle 2D class averages indicated regular axial spacings of 4.9 Å, which were also visible in images of class averages (Supplemental Fig. 2a, b). 2D class averages and iterative 3D classifications converged on a single core morphology to yield a 3.0 Å resolution map of the fibril core (Fig. 1b, c; Supplemental Fig. 2c and Supplemental Table) with stacked rungs occurring perpendicular to the fibril axis (Fig. 1c, f).

**Fig. 2:**
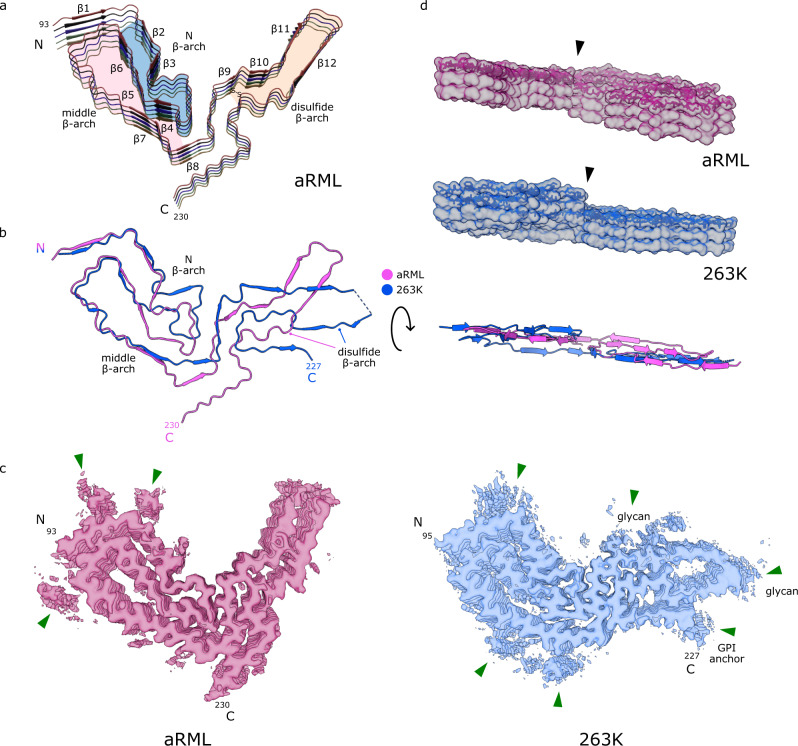
Comparison of aRML and 263K prions. **a** Ribbon diagram of aRML core (stack of 5), with structural motifs as colored. **b** Overlay of aRML and 263K cores. **c** Contour EM density maps of aRML and 263K. Green arrows indicate peripheral unassigned densities associated with cationic residues in the N-terminal lobes of both strains, but absent in the aRML C-terminal lobe, consistent with aRML’s lack of glycans and glycolipid anchors. **d** Lateral views of aRML and 263K EM maps (stacks of 3) with stick models embedded in top rung. Arrowhead indicates protrusion of anvil-like head of the N β-arch. Underneath is a ribbon overlay of aRML and 263K monomers indicating differences in planarity.

We threaded the polypeptide comprising the aRML protease-resistant core into the cryo-EM density map, with iterative real and Fourier space refinements and validation as indicated in Supplemental Table 1. In silico conformational sampling produced a backbone RMSD of 2.2 Å demonstrating the relative stability of the native state model (see the “Methods” section, Supplemental Fig. 3). The resolved aRML structure comprises residues 93–230 (Fig. 1d). Some peripheral electron densities were not assigned to any of these residues (Fig. 2c, green arrows) and are assumed to reflect either tightly bound remnants of more extreme N-terminal PrP sequence after partial proteolysis, or unidentified non-PrP ligands. Peripheral densities that we previously observed adjacent to the C-terminus and N-linked glycosylation sites in the 263K prion structure^1^ are absent in the aRML density map (Fig. 2c). As with 263K, the PrP monomers span the entire fibril cross-section and are stacked parallel and in-register perpendicular to the fibril axis with an average spacing of 4.9 Å (Fig. 1f). As detailed below, several major features of the aRML structure are analogous to, but distinct from, those in the 263K structure (Fig. 2)^1^.

Residues 95–104 near the N-terminus of the aRML core sequence are held tightly against residues 140–144 by an interdigitation of alternate sidechains across the interface (Fig. 2c and Supplemental Fig. 4a). This steric zipper interface is similar to that seen for analogous residues in 263K^1^. In both aRML and 263K, the backbones of these sequences are not precisely coplanar within a given monomer but are staggered slightly with respect to one another axially.

As in 263K, in aRML a β-arch of residues ~103–140 extends from the N-terminal steric zipper residues and has an anvil-like head that we previously, and unaptly^12^, described as a “Greek key” (Fig. 2a and Supplemental Fig. 4b)^1,12^. However, despite having an identical sequence within residues 112–137 (mouse numbering), the heads of this N-proximal (N) β-arch in aRML and 263K have markedly different conformations (Fig. 2b). A notable example of this is the sidechain of Y127, which is on the inside of the anvil in aRML, while the analogous Y128 of 263K is on the outside^1^.

The C-terminal flank of the N β-arch provides a flank of an overlapping β-arch of residues ~124–167 (Fig. 2a) to form a middle β-arch. The tip of this arch forms the steric zipper with residues 95–104 described above (Supplemental Fig. 4a). Although aRML and 263K both have middle β-arches, they differ in sequence at residues 138 and 154 (mouse numbering) and in the conformation of residues 125–132 in particular (Fig. 2b). Also, the gap between the N- and C-terminal flanks of this arch is wider, and presumably more hydrated, in aRML (Supplemental Fig. 4e).

A staggered interface between the head of the N β-arch and the C-terminal half of the core has the sidechains of residues 120–124 of a given monomer interacting with sidechains of residues 169–176 of the both the same monomer and the one below it. While this stagger is less pronounced than that of 263K^1^, it contributes to deviations from planarity of each monomer (Fig. 2d).

The β-arch defined by the disulfide bond between C178 and C213 is stabilized by a tight interface (Supplemental Fig. 4c). This occurs before a bend and modest widening of the β-arch to encompass a presumably hydrated pocket (Supplemental Fig. 4d), which is much wider in 263K (Fig. 2c)^1^.

Another striking difference between the aRML and 263K structures is the orientation of the C-terminal residues that, in 263K, provide a linkage to the GPI anchor. In aRML, the residues 220–230 project away from the disulfide arch and interface with residues 165–170 (Fig. 2a, b). In contrast, the C-terminal residues of 263K associate with the disulfide arch^1^.

## Discussion

Comparison of the aRML structure with the 263K prion structure^1^ reveals that these two ex vivo rodent prion strains both have PIRIBS amyloid architectures with several similar structural elements. However, the detailed conformations and relative orientations of these and other elements differ markedly, providing distinct conformational templates for incoming monomers as proposed previously^1,14^. Also, the amino acid sequences differ at 8 residues within the ordered cores of aRML and 263K^1^; presumably contributing to the respective templating activities, and, hence, species specificities of these prion strains. The extent to which the conformational differences between 263K and RML prions are dictated by sequence differences versus conformational templating remains to be determined. In both strains, the opposite ends of the fibrils are not equivalent. Among the key differences is the non-coplanarity of the anvil-like head of the N β-arch which protrudes at one end and recedes at the other (Figs. 1f and 2d). Such non-equivalence presumably affects the relative mechanism and kinetics of PrP conversion at the fibril ends.

aRML prions lack GPI anchors, but are still ultimately lethal^7^. Thus, although GPI anchors and glycosylation affect the neuropathological lesions observed in infected animals (e.g. refs. ^6,7,15–17^), neither is an essential prerequisite for neuropathogenicity. From a structural perspective, these post-translational modifications do not seem to substantially alter the core structures of at least three murine prion strains, as probed by infrared spectroscopy^5^. Moreover, in vivo passages of RML scrapie from wildtype to anchorless PrP transgenic mice and back again do not significantly alter the incubation period or clinical manifestations of the strain in wildtype mice^7^. However, such a passage history was reported to result in stable changes in susceptibility to inhibition of prion infection by swainsonine and other inhibitors in a cell panel assay^18^. These results implied that subtle alterations in the RML prion core conformation occurred in the anchorless transgenic mice that were faithfully propagated in subsequent passages in wildtype mice.

Of note, a structure in an accompanying manuscript by Manka and colleagues for wildtype (wt, i.e., GPI-anchored and more glycosylated) RML fibrils appears to be quite similar overall to that of aRML^12^. However, whereas the ordered core of aRML spanned residues 93–230, the wtRML core spanned residues 94–225. The less-ordered extreme C-terminus in the wtRML structure likely relates to the presence of the GPI anchor, which is absent on aRML fibrils. Also, whereas our analysis of the aRML model identified 12 β-sheets, the wtRML model indicated 15 β-sheets. This variance may relate to the differences in the poorly resolved post-translational modifications and, potentially, non-PrP ligands between these distinct RML prion preparations. Alternatively, the cryo-EM density maps used to derive the atomic models may not be sufficiently resolved in all areas of the fibril cores to unambiguously pinpoint every polypeptide backbone torsional angle and the corresponding secondary structure. Nonetheless, the predominant similarity between the aRML and wtRML core structures, together with the retention of most strain properties through passages into anchorless PrP transgenic mice and then back into wildtype mice, is consistent with the core structure providing the primary “code” for the fundamental properties of a prion strain. However, strain phenotypes can be further modulated markedly by these post-translational modifications, or lack thereof, in a given type of host^6,7,15–17,19–22^.

In any case, the comparisons of RML prions to 263K prions illustrate structural permutations that discriminate these two brain-derived prion strains, but much further work is needed to characterize the full spectrum of mammalian prion structures and the modulating influences of GPI anchors and glycans.

## Methods

### PrP^Sc^ fibril purification and bioassay

All mice were housed at the Rocky Mountain Laboratory in an AAALAC accredited facility in compliance with guidelines provided by the Guide for the Care and Use of Laboratory Animals (Institute for Laboratory Animal Research Council). Experimentation followed Rocky Mountain Laboratory Animal Care and Use Committee approved protocols 2018‐011, 2016‐039, or 2021-011-E.

aRML (also known as Chandler) prion strain fibrils were purified from brains of transgenic mice expressing only GPI-anchorless PrP and characterized as part of previous studies^5,10^. Briefly, brain homogenates were treated with a 2% sarkosyl buffer and benzonase (EMD Millipore) to digest nucleic acids and then ultracentrifuged to pellet PrP^Sc^. The pellet was resuspended, treated with proteinase K (PK), a high salt (1.7 M NaCl) and 30 mM EDTA buffer, and centrifuged through a sucrose cushion containing 0.5% sulfobetaine 3–14. The pellet was washed in 0.5% sulfobetaine 3–14, pelleted, and resuspended using cuphorn sonication into 0.5% sulfobetaine 3–14 in 20 mM sodium phosphate, 130 mM NaCl; pH 7.4. Further characterizations of such aRML preparations have been described previously^5^. Additional fibril manipulations prior to cryo-EM grid preparation were performed as previously reported^1^. Briefly, fibril preparations were vortexed and allowed to sit for several minutes to pellet highly bundled fibrils. Aliquots from the supernatant fraction were diluted in 20 mM Tris pH 7.4, 100 mM NaCl containing 0.02% amphipol 8–35 and sonicated immediately prior to grid preparation.

To estimate the infectivity of the purified aRML preparation, male tga20 homozygous mice^11^ were anesthetized with isoflurane and injected in the left-brain hemisphere with 10-fold serial dilutions beginning with 100 ng of purified anchorless RML prep diluted in 30 µl phosphate buffered balanced saline solution + 2% fetal bovine serum. Following inoculation, mice were monitored for onset of prion disease signs and euthanized when they displayed signs of prion disease including ataxia, flattened posture, delayed response to stimuli, and somnolence.

### Cryo-EM grid preparation

C-Flat 1.2/1.3 300 mesh copper grids (Protochips, Morrisville, NC) were glow-discharged with a 50:50 oxygen/hydrogen mixture in a Solarus 950 (Gatan, Pleasanton, CA) for 10 s. Grids were mounted in an EM GP2 plunge freezer (Leica, Buffalo Grove, IL) and a 3 μl droplet of 0.02% amphipol A8-35 in phosphate buffered saline was added to the carbon surface and hand blotted to leave a very thin film. The tweezers were then raised into the chamber of the plunge freezer, which was set to 22 °C and 90% humidity. 3 μl of recently sonicated sample was added to the carbon side of the grid and allowed to sit for 60 s. The sample was subsequently blotted for ∼4 s followed by a 3 s drain time before plunge freezing in liquid ethane kept at −180 °C. Grids were mounted in AutoGrid assemblies.

### Cryo-electron tomography

For tomography, grids were prepared as above except that 5 nm Protein A gold (CMC, Utrecht, The Netherlands) was added for fiducial markers. The grid assemblies were loaded into a Krios G1 (Thermo Fisher Scientific, Waltham, MA) transmission electron microscope operating at 300 kV with a K3 (Gatan, Pleasanton CA) and a Biocontinuum GIF (Gatan, Pleasanton CA) with a slit width of 20 eV. Tilt series were acquired using SerialEM^23^ at a 0.45 Å pixel size at ±60°, 2° increment in a dose symmetric manner around 0°^24^ with defocus values ranging from −3 to −6 μm and a total dose of ~60 e^−^/Å^2^. Tomograms were reconstructed and 12 were analyzed using IMOD^25^. To verify that our imaging system preserved handedness, we negatively stained bacteria onto a finder grid and acquired tilt-series of an asymmetric letter to confirm orientation did not change during imaging nor through tomographic reconstruction^26^. We then used the bacteria as fiducials to confirm that there were only rotation changes during magnification increases from the tilt-series magnification of the finder grid letter to the tilt-series magnification of aRML.

### Image acquisition and processing for helical reconstruction

Initial map and helical twist parameters were generated based on our recently published dataset^1^, as well as on cryo-electron tomographic analyses of aRML fibrils described above. Motion correction of raw movie frames was performed with RELION 3.1^13^. CTF estimation was performed using CTFIND4.1^27^. Fibrils were handpicked then extracted using large and small box sizes. The longer helical segments were extracted with a box size of 1280 pixels and were downscaled to a box size of 256 pixels. The shorter segments were extracted at 400 pixel box size. 2D classes, from the long segments were used to estimate the cross-over distance of the fibril for estimating initial twist parameters. 2D classes from the short segments were used to generate an initial 3D model.

Higher resolution data was collected using a Titan Krios G3i (Thermo Fisher Scientific, Waltham, MA) with a K3 camera and BioQuantum GIF (Gatan, Pleasanton, CA) with images acquired at 0.55 Å/pixel at Super Resolution mode, 60 e^−^/Å^2^, and 60 total frames. Movies were motion corrected and the CTF estimated as above. Fibrils were picked manually and segments were extracted with an inter-box distance of 14.6 Å using box size of 740 pixels that was down sampled to 370 pixels. Reference-free 2D class averaging was performed, using a regularization parameter of *T* = 2, a tube diameter of 180 Å, and the translational offset limited to 4.8 Å. The initial model was used for 3D auto refinement with C1 symmetry, initial resolution limit of 40 Å, initial angular sampling of 3.7°, offset search range of 5 pixels, initial helical twist of −0.72°, initial helical rise of 4.85 Å, and using 50% of the segment central *Z* length. The output from auto refinement was used for 3D classification without allowing for image alignment to remove poorly aligned segments from auto refinement. Classes were selected for further refinement based on similarity of features in their cross-section (excluding visually low resolution and poorly aligned classes), estimated resolution, overall accuracy of rotation and translation, and Fourier completeness. Auto-refinement was then performed while optimizing the helical twist and rise, yielding a final map with a twist of −0.637° and rise of 4.876 Å. Iterative cycles of CTF refinement, Bayesian polishing, and auto refinement were used until resolution estimates stabilized. Post processing in RELION was performed with a soft-edged mask representing 10% of the central *Z* length of the fibril. Resolution estimates were obtained between independent refined half-maps at 0.143 FSC.

### Model building

De novo building of an aRML atomic model was conducted using Coot^28^, with the assumption that residues comprising the protease-resistant core (i.e. ~90–231) were included in the amyloid core. Individual subunits were translated to generate a stack of five consecutive subunits, and translated subunits rigid-body fit in Coot. Iterative real-space refinement and validation with Coot and Phenix^29,30^ were performed, with Fourier space refinements being conducted using RefMac5. Model validation was performed with CaBLAM^31^, MolProbity^32^, and EMringer^33^, and any outliers/clashes identified and corrected with subsequent iterative refinements/validation. Model renderings in Figs. 1 and 2 were performed using Chimera X.

### Molecular dynamics simulations

Molecular dynamics simulations were performed using NAMD 2.14^34^ with the CHARMM36 forcefield^35^. After the addition of protons utilizing the HBUILD functionality in the CHARMM molecular dynamics platform^36^, a disulfide bond between cysteines 178 and 213 was generated with the use of the DISU patch. The individual monomer chains in the fibril were capped using the ACE and CNEU patches. The system was then solvated with TIP3P water molecules in a cubic box containing neutralizing Na^+^ and Cl^−^ ions. A gradient of backbone and sidechain restraints ranging from 10 kcal/mol/ Å^2^ to 1 kcal/mol/ Å^2^, was utilized in iterative runs of conjugate gradient minimization and were subsequently removed in the last 15,000 steps. A 1 ns NVT equilibration simulation with backbone restraints was performed at a temperature of 300 K maintained with Langevin dynamics. The simulation advanced at a timestep of 1 fs and the particle mesh Ewald algorithm was used to calculate long-range electrostatics. Non-bonded interactions had a cutoff of 10 Å and the rigid bond algorithm was applied to all bonds containing hydrogen atoms. Subsequently, a 1 ns NPT equilibration was performed with backbone restraints followed by a 10 ns production run which advanced at a 2 fs timestep. Pressure was kept constant using the Langevin-piston method. After 10 ns, the restraints were removed, and the simulation continued for an additional 170 ns. The trajectories were analyzed using VMD^37^ and Bio3D^38^.

### Reporting summary

Further information on research design is available in the Nature Research Reporting Summary linked to this article.

## Supplementary information

Supplementary Information

Reporting Summary

## Data Availability

Cryo-EM density maps and the atomic model of PrP^Sc^ fibrils have been deposited at the Electron Microscopy Data Bank and Protein Data Bank with accession codes EMD-25824 and PDB ID 7TD6, respectively. The purified aRML prions, and the brains from which they are isolated, are extremely limited. While we might be able to share small amounts of these materials upon request, we cannot guarantee availability. However, the Tg44 anchorless PrP transgenic mice, and RML scrapie inocula are more readily available upon request.
