# Supplementary material for: Cryo-EM structure of anchorless RML prion reveals variations in shared motifs between distinct strains
Source: Nat Commun. 2022 Jul 13;13:4005. doi: 10.1038/s41467-022-30458-6 (PMC9279418; doi:10.1038/s41467-022-30458-6)
Supplement: Supplementary file 1 — Supplementary Information [file 41467_2022_30458_MOESM1_ESM.pdf]

<u>Supplementary Information for</u>:

Cryo-EM structure of anchorless RML prion reveals variations in shared motifs between distinct strains

Forrest Hoyt, Heidi G. Standke, Efrosini Artikis, Cindi L. Schwartz, Bryan Hansen, Kunpeng Li, Andrew G. Hughson, Matteo Manca, Olivia R. Thomas, Gregory J. Raymond, Brent Race, Gerald S. Baron, Byron Caughey and Allison Kraus

Supplemental Table

	aRML (PDB ID: 7TD6, EMDB ID: EMD-25824)
**Data collection and processing**	
Magnification	81,000x
Voltage (kV)	300
Electron dose (e-/ Å^2)	60
Pixel size (super resolution)	0.545
Symmetry imposed	C1
Initial particle segments	135939
Final particle segments	15857
Map resolution (Å) (FSC 0.143)	3.03
Helical rise (Å)	4.876
Helical twist (°)	-0.637
Map sharpening *B* factor (Å^2)	9.28
**Model Refinement**	
Initial Model	*de novo*
R.M.S. deviations	
Bond lengths (Å)	0.002
Bond angles (°)	0.570
MolProbity score	1.43
Clash score	7.94
Rotamer outliers (%)	0
Ramachandran plot	
Favored (%)	100.00
Allowed (%)	0
Outliers (%)	0
EM Ringer score	2.90
Model vs. Data (CC)	0.74

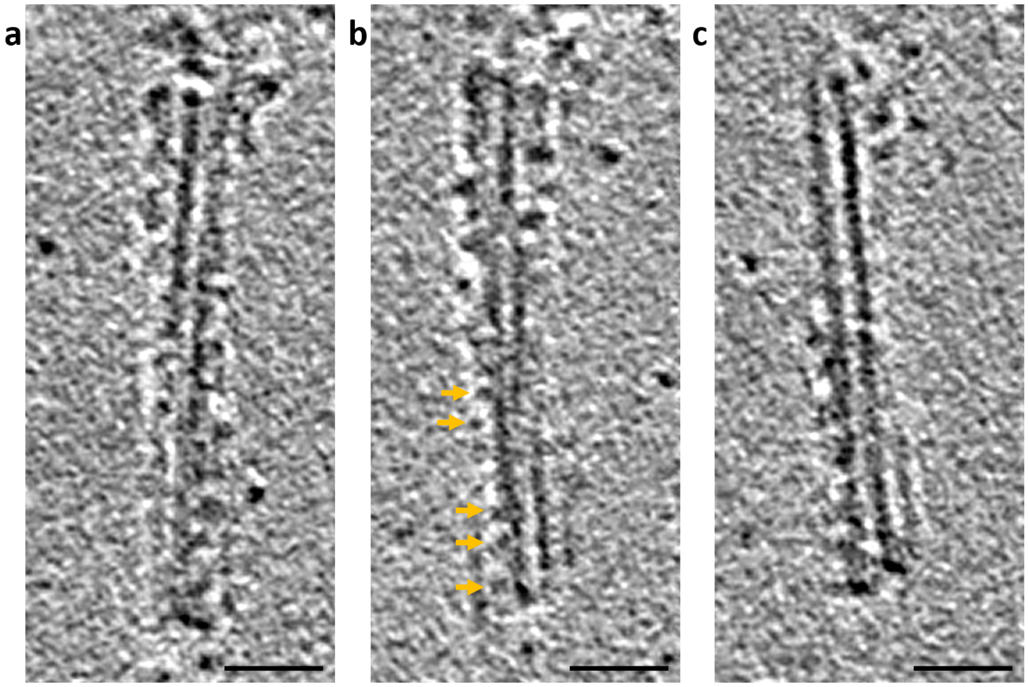

Supplemental Fig. 1. Cryo-electron tomography of a representative fibril with globules (arrows). The left-handed twist is evident when visualizing bottom (**a**), middle (**b**), and top (**c**) tomographic slices (4.5 nm thick). Scale bars = 25 nm.

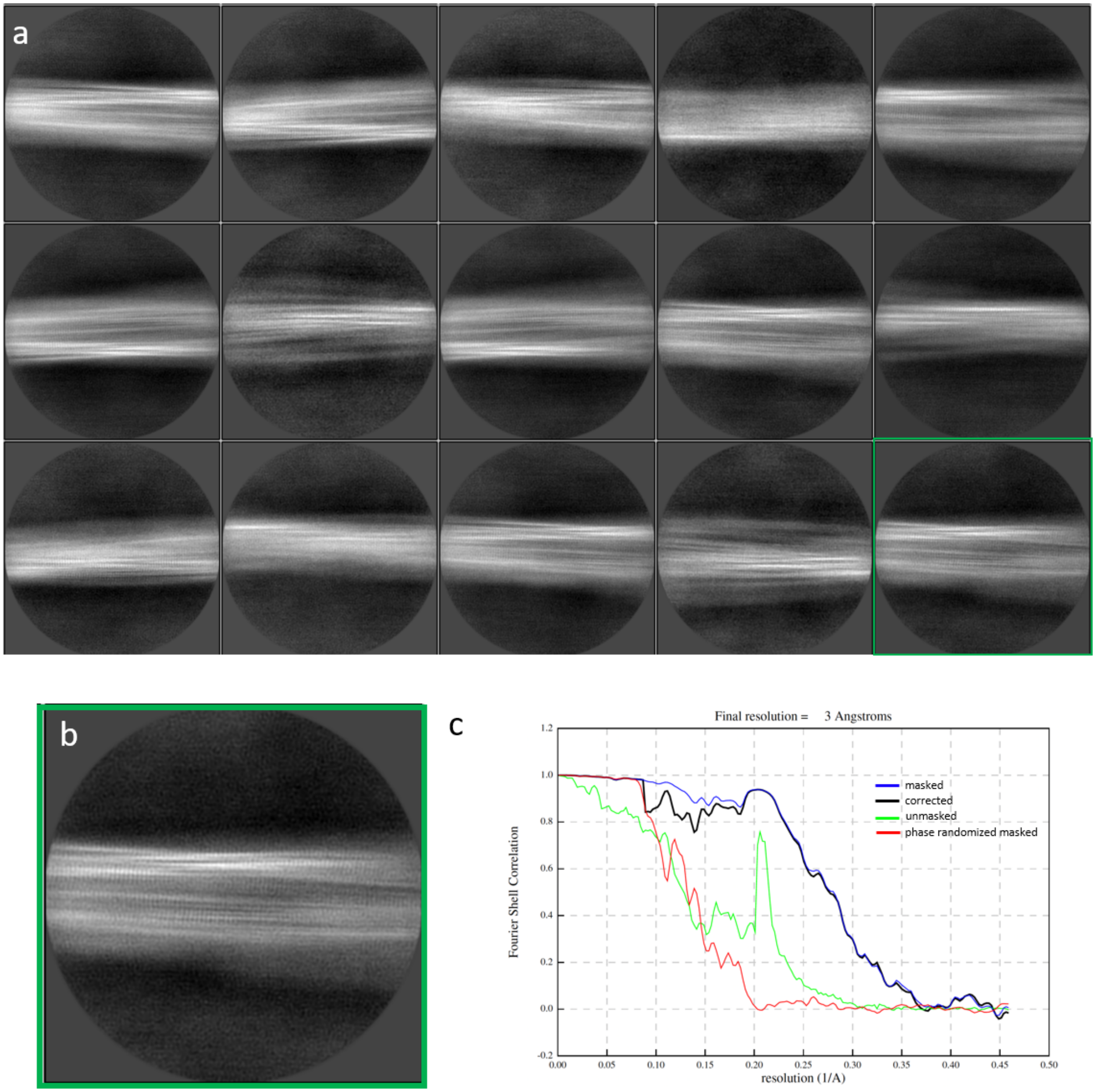

Supplemental Fig. 2. **a**. Representative 2D class averages depicting lateral views of aRML fibril segments (29 classes shown of 50 total used to reconstruct 3D density map). **b**. Enlarged view of one of the 2D classes (highlighted in green in **a**) showing the 4.9Å repeated spacing perpendicular to the fibril axis. **c**. Fourier shell correlation plots of masked and unmasked models.

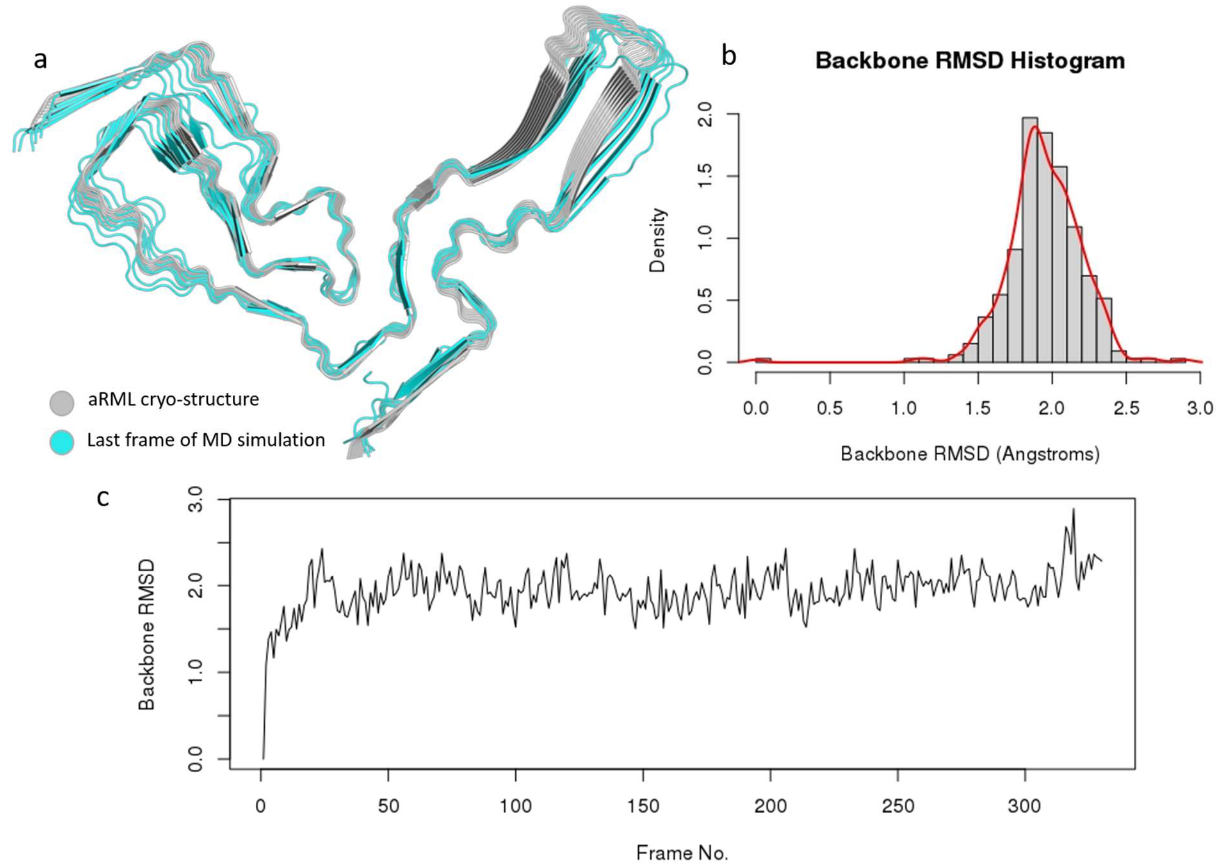

Supplemental Fig. 3. a. Overlay of the aRML cryo-structure (shown in grey) and the last frame of a 180 ns NPT molecular dynamics simulation (shown in cyan) **b.** The backbone RMSD was computed for the last 170 ns of the production run after the removal of backbone restraints and utilizes the initial frame as a point of reference. The normalized histogram depicts the distribution of backbone RMSD values of the sampled conformations. The average backbone RMSD is 2.2 Å **c.** The backbone RMSD is plotted as a function of time displayed in 0.5 ns intervals indicating convergence. Similar results were obtained from 2 additional independent simulations of comparable length.

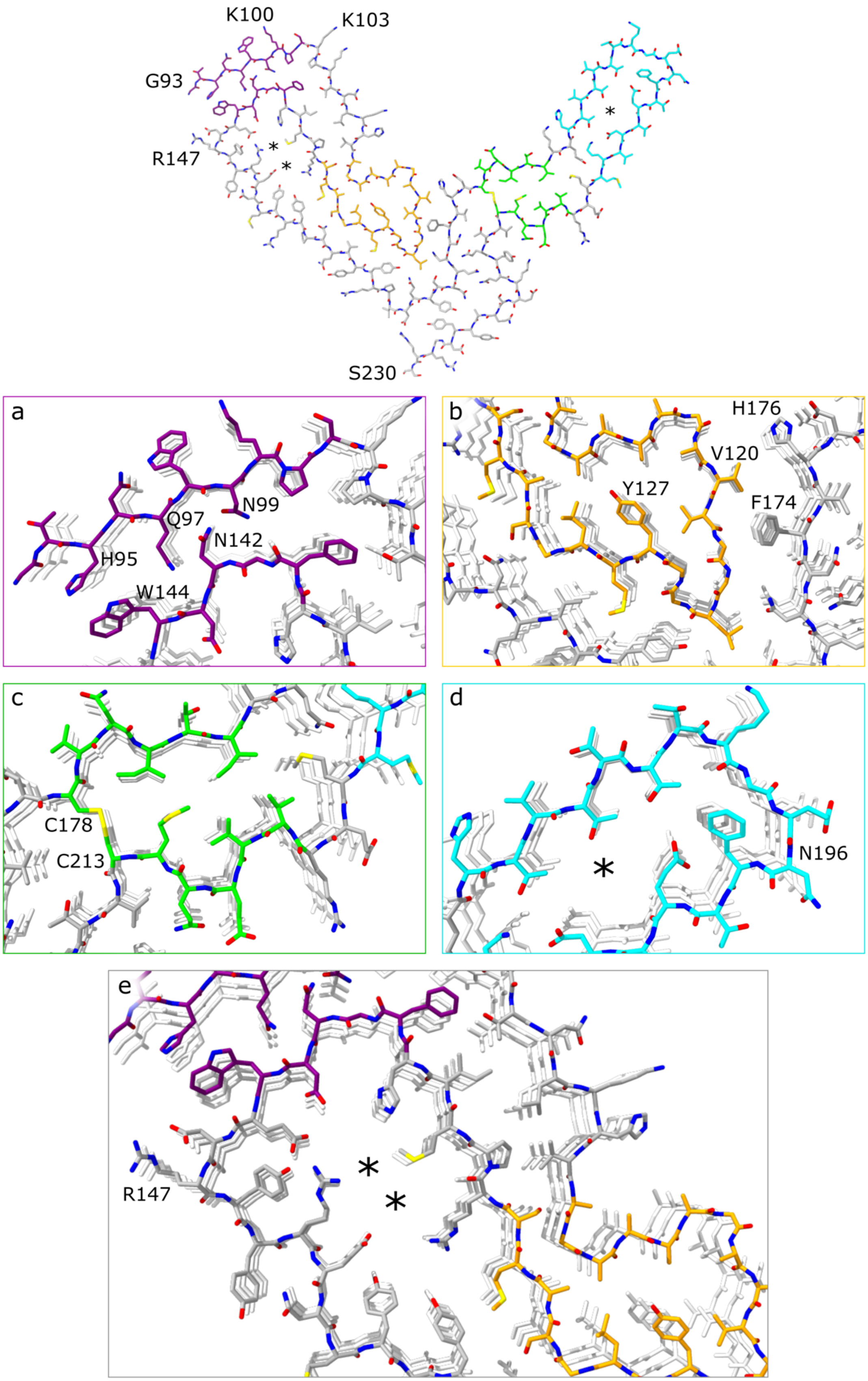

K100
K103
G93
R147
*
*
*
S230
a
N99
Q97
N142
H95
W144
b
H176
V120
Y127
F174
c
C178
C213
d
*
N196
e
*
*
R147

Supplemental Fig. 4. Features of the aRML prion core. **a**, Steric zipper (colored in purple) is formed by the N-terminus and tip of the mid β-arch, inclusive of residues H95, Q97, N99, N142, W144. **b**, the head of the N β-arch (tan) comprised of hydrophobic residues occurs adjacent to the central strand, stabilized by hydrophobic interface of V120, F174, and H176 residues. **c**, Disulfide bond (C178 – C213) forms the base of disulfide β-arch (green) that is stabilized by a tight interface adjacent to a widening of the arch **(d)**, giving a presumably hydrated pocket (turquoise) marked with an asterisk. **e**, A second hydrated gap occurs in the mid β-arch (grey) of the aRML core, also marked with asterisks.
