# Supplementary material for: Cryo-EM structure of anchorless RML prion reveals variations in shared motifs between distinct strains
Source: Nat Commun. 2022 Jul 13;13:4005. doi: 10.1038/s41467-022-30458-6 (PMC9279418; doi:10.1038/s41467-022-30458-6)
Supplement: Supplementary file 2 — Reporting Summary [file 41467_2022_30458_MOESM2_ESM.pdf]

nature portfolio

Corresponding author(s): Byron Caughey, Allison Kraus

Last updated by author(s): May 25, 2022

Reporting Summary

Nature Portfolio wishes to improve the reproducibility of the work that we publish. This form provides structure for consistency and transparency in reporting. For further information on Nature Portfolio policies, see our Editorial Policies and the Editorial Policy Checklist.

Statistics

For all statistical analyses, confirm that the following items are present in the figure legend, table legend, main text, or Methods section.

n/a	Confirmed	
☐	☒	The exact sample size (n) for each experimental group/condition, given as a discrete number and unit of measurement
☐	☒	A statement on whether measurements were taken from distinct samples or whether the same sample was measured repeatedly
☒	☐	The statistical test(s) used AND whether they are one- or two-sided<br>*Only common tests should be described solely by name; describe more complex techniques in the Methods section.*
☒	☐	A description of all covariates tested
☒	☐	A description of any assumptions or corrections, such as tests of normality and adjustment for multiple comparisons
☒	☐	A full description of the statistical parameters including central tendency (e.g. means) or other basic estimates (e.g. regression coefficient) AND variation (e.g. standard deviation) or associated estimates of uncertainty (e.g. confidence intervals)
☒	☐	For null hypothesis testing, the test statistic (e.g. F, t, r) with confidence intervals, effect sizes, degrees of freedom and P value noted<br>*Give P values as exact values whenever suitable.*
☒	☐	For Bayesian analysis, information on the choice of priors and Markov chain Monte Carlo settings
☒	☐	For hierarchical and complex designs, identification of the appropriate level for tests and full reporting of outcomes
☒	☐	Estimates of effect sizes (e.g. Cohen's d, Pearson's r), indicating how they were calculated

Our web collection on statistics for biologists contains articles on many of the points above.

Software and code

Policy information about availability of computer code

Data collection	Serial EM 3.8.0
Data analysis	Relion 3.1, CTFIND4.1, Coot0.9.2-pre,0.9.6, Chimera X 1.2.5, RefMac5, Phenix 1.19-4092,1.19.2-4158(withCaBLAM, MolProbity, EMringer) NAMD 2.14, CHARMM c45b1, VMD 1.9.3, Bio3D 2.4-2, IMOD4.10.48.37755

For manuscripts utilizing custom algorithms or software that are central to the research but not yet described in published literature, software must be made available to editors and reviewers. We strongly encourage code deposition in a community repository (e.g. GitHub). See the Nature Portfolio guidelines for submitting code & software for further information.

Data

Policy information about availability of data

All manuscripts must include a data availability statement. This statement should provide the following information, where applicable:

- Accession codes, unique identifiers, or web links for publicly available datasets
- A description of any restrictions on data availability
- For clinical datasets or third party data, please ensure that the statement adheres to our policy

Cryo-EM density maps and the atomic model of PrPSc fibrils have been deposited at the Electron Microscopy Data Bank and Protein Data Bank with accession codes EMD-25824 and PDB ID 7TD6, respectively.

Field-specific reporting

Please select the one below that is the best fit for your research. If you are not sure, read the appropriate sections before making your selection.

☒ Life sciences ☐ Behavioural & social sciences ☐ Ecological, evolutionary & environmental sciences

For a reference copy of the document with all sections, see nature.com/documents/nr-reporting-summary-flat.pdf

Life sciences study design

All studies must disclose on these points even when the disclosure is negative.

Sample size	aRML prion fibrils were derived from pooled brain tissue of 4-5 animals for subsequent cryo-EM imaging and analysis. 2272 movies were collected, that were distilled to 1123 after CTF estimation for particle picking. Relion 3.1 workflow was used to determine the 3.0 Å map (inclusive of 15857 final particle segments from an initial particle segments of 135939).<br><br>Tomographic analyses:<br>12 tomograms were analyzed to establish handedness of aRML fibrils (n=64).<br><br>Three replicate molecular dynamics trajectories were performed on the final molecular model.<br><br>Serial dilutions of the aRML fibril preparation was inoculated into groups of 4 or 6 tga20 mice (see Methods). Then RML prion infected brain homogenate was inoculated into 7 tga20 mice.
Data exclusions	Particles segments distilled as per the standard and published Relion 3.1 workflow of data processing. See methods for image processing of aRML fibrils. Particle picking involved selection of any visible and apparently individual fibril segment, without exclusion of any particle morphology. Reference-free 2D class averaging was used to distill the data set to achieve sufficient quality to yield a 3.0Å resolution map.
Replication	The cryo-EM aRML structure reported in this study is consistent with the lower resolution structure determined in our previous study (Kraus et al., Mol Cell 2021). Overall, aRML fibril morphologies observed were consistent over multiple independent grid preparations for this study. Sufficient ice quality for high resolution imaging was determined with grid screening, and 2272 movies collected on the highest quality grid. 12 independent tomograms were used to determine that all fibrils analyzed (n=64) were left-handed. Three independent molecular dynamics trajectories were performed. The same fibril preparation used for cryo-EM analysis was assessed by bioassay, with a starting inoculation of 100 ng and subsequent 100-fold dilutions with 4-6 animals per group.<br>Notwithstanding grid preparations of insufficient quality for high resolution cryo-EM as described above, all attempts at replication were successful.
Randomization	Cryo-EM analysis requires screening of grids for appropriate sample quality to yield high resolution data, with exclusion of areas with non-vitreous or thick ice. After this necessary procedural exclusion, randomization is not applicable to the study with automated movie aquisition and data analysis being conducted using mathematical and unbiased (reference-free) class averaging.
Blinding	With regards to the end-point dilution prion bioassays used in this study, the final discrimination of diseased versus healthy animals is made only when the clinical signs of terminal prion disease are unmistakable and essentially non-subjective, making blinding of little value or consequence. Blinding is not applicable to the Cryo-EM data acquisition and data processing as the identity of the sample is required towards determining a de novo structure.

Reporting for specific materials, systems and methods

We require information from authors about some types of materials, experimental systems and methods used in many studies. Here, indicate whether each material, system or method listed is relevant to your study. If you are not sure if a list item applies to your research, read the appropriate section before selecting a response.

Materials & experimental systems

n/a	Involved in the study	
☒	☐	Antibodies
☒	☐	Eukaryotic cell lines
☒	☐	Palaeontology and archaeology
☐	☒	Animals and other organisms
☒	☐	Human research participants
☒	☐	Clinical data
☒	☐	Dual use research of concern

Methods

n/a	Involved in the study	
☒	☐	ChIP-seq
☒	☐	Flow cytometry
☒	☐	MRI-based neuroimaging

Animals and other organisms

Policy information about studies involving animals; ARRIVE guidelines recommended for reporting animal research

Laboratory animals	Both male and female tg44+/+ transgenic mice expressing GPI-anchorless PrP were inoculated as weanlings with the RML strain of scrapie and brain tissue harvested when the animals were sick at ages of ~ 300-400 days. Mice were housed in a controlled 12 hour light/dark cycle with ambient temperature of 70F +/- 2 degrees. Humidity was controlled and typically measured between 40-50%.
Wild animals	No wild animals were used in the study.
Field-collected samples	No field-collected samples were used in the study.
Ethics oversight	Mice were housed at RML in an AAALAC-accredited facility. Experiments were in accordance with the NIH RML Animal Care and Use Committee approved protocols (2018-011, 2016-039, 2021-011-E).

Note that full information on the approval of the study protocol must also be provided in the manuscript.
